# Reference intervals of serum hyaluronic acid corresponding to the radiographic severity of knee osteoarthritis in women

**DOI:** 10.1186/1471-2474-14-34

**Published:** 2013-01-18

**Authors:** Haruka Kaneko, Muneaki Ishijima, Tokuhide Doi, Ippei Futami, Lizu Liu, Ryo Sadatsuki, Anwarjan Yusup, Shinnosuke Hada, Mitsuaki Kubota, Takayuki Kawasaki, Yoshitomo Saita, Yuji Takazawa, Hiroshi Ikeda, Hisashi Kurosawa, Kazuo Kaneko

**Affiliations:** 1 Department of Medicine for Orthopaedics and Motor Organ, Juntendo University Graduate School of Medicine, Tokyo, Japan; 2 Department of Orthopaedics, Faculty of Medicine, Juntendo University, Tokyo, Japan; 3 Sportology Center, Juntendo University Graduate School of Medicine, Tokyo, Japan; 4 Fukuoka Clinic, Tokyo, Japan; 5 Department of Orthopaedic Surgery, Juntendo Tokyo Metropolitan Koto Geriatric Medical Center, Tokyo, Japan

**Keywords:** Osteoarthritis (OA), Hyarulonic Acid (HA), Reference intervals, Biomarker, Radiography

## Abstract

**Backgroud:**

While serum levels of hyarulonic acid (sHA) is known to be useful for a burden of disease biomarker in knee OA, it is far from practical. The reference intervals must be established for biomarkers to be useful for clinical interpretation. The aim of this study was to establish the reference intervals of sHA corresponding to the radiographic severity of knee OA for elucidating whether sHA can be useful as a burden of disease marker for individual patient with knee OA.

**Methods:**

372 women with Kellgren & Lawrence grade (K/L) 1 through 4 painful knee OA were enrolled in this study. The patients included 54 with K/L 1, 96 with K/L 2, 97 with K/L 3, and 118 with K/L 4. Serum samples were obtained from all subjects on the day that radiographs taken. A HA binding protein based latex agglutination assay that employed an ELISA format was used to measure sHA. Age and BMI adjusted one way ANOVA was used to set the reference intervals of sHA.

**Results:**

The reference intervals for sHA corresponding to the patients with K/L 4 (49.6 – 66.5 ng/ml) was established without any overlap against to those with K/L 1, 2 and 3, while those with K/L 1, 2 and 3 showed considerable overlap.

**Conclusions:**

These results indicate that sHA can be available as a burden of disease marker for the individuals with severe knee OA (K/L 4), while it is not for those with primary to moderate knee OA (K/L 1–3).

## Background

Osteoarthritis (OA) Biomarkers Network, which was funded by National Institute of Health (NIH) and developed as a partnership with Osteoarthritis Research Society International (OARSI) and the Arthritis Foundation, proposed the BIPEDS biomarker classification (Burden of disease, Investigative, Prognostic, Efficacy of intervention, Diagnosis of the Disease and Safety of interventions) which suggests the optimal study design and analytic methods for use in OA investigations [1,2]. Burden of disease markers assess the severity of disease within a particular joint among individuals with OA typically at a single point in time. Studies of burden of disease markers require comparison with one or more gold standard methods, such as radiography, of determining disease severity [3].

The serum level of hyaluronic acid (sHA) is a potential biomarker for the establishment of a proper management system in knee OA. Although sHA has been reported to be a useful burden of disease marker in OA [4-7], it is still far from being practical.

The reference intervals for a biomarker must be established before the marker can be adequate and useful for clinical interpretation. The International Federation of Clinical Chemistry and Laboratory Medicine (IFCC) and Clinical and Laboratory Standards Institute (CLSI) have defined reference intervals as the interval between two reference limits. In most cases, reference intervals are designated as the interval between and including two numbers, an upper and lower reference limit, which are estimated to enclose a specified percentage (usually 95%) of the values for a population from which the reference subjects are drawn [8].

The purpose of this study was to establish the reference intervals of sHA corresponding to the radiographic severity of knee OA in order to allow sHA to become a more practical biomarker in knee OA.

## Methods

This prospective cohort study protocol was approved by the institutional review board of Juntendo University and conducted in accordance with the Declaration of Helsinki. All patients provided their written informed consent before enrollment in this trial. Three-hundred seventy-six women ranging in age from 48 to 86 years (mean 69.1) with knee pain were recruited. Patients with knee pain complained pain in the medial femorotibial compartment of the studied knee on most days of the month prior to examination and removal of body fluids. None of the patients had experienced any traumatic episodes during this period. They fulfilled the criteria of knee OA of the medial femorotibial joint as defined by the American College of Rheumatology (ACR) criteria [9]. As several other factors, such as impaired renal, hepatic function and corticosteroid usage, affect serum HA levels, patients who had these diseases were excluded. Standing, extended and antero-posterior view, and lateral and skyline view radiographs were taken at the first visit. The staging of knee OA based on radiographic examination was assessed using Kellegren and Laurence (K/L) grading. The patients included 54 with K/L grade 1, 96 with K/L grade 2, 97 with K/L grade 3, and 118 with K/L grade 4 based on the weight-bearing antero-posterior radiographs.

Serum samples were obtained from all subjects on the day that radiographs taken. As there is a diurnal variation in HA levels [10,11], fasting blood samples were collected at late morning-early afternoon for serum analyses. The serum samples were stored at -80°C until analysis. sHA was measured using an HA binding protein based latex agglutination assay (Chugai Diagnostics, Tokyo, Japan) that employed an ELISA format and the intra-assay and inter-assay variation were less than 5%.

The analyses were performed using SPSS ver.17 (SPSS Inc., Chicago, IL). The normality of the distribution of each group was tested by Kolmogorov-Smirnoc statistics. A correlation analysis was conducted by Spearman’s correlation coefficient. Parametric comparisons were necessary for the age and body mass index (BMI) adjusted one way analysis of variance (ANOVA). However, as sHA levels of the patients did not show normal distribution, they were logarithmically transformed (ln-sHA) and the age and BMI adjusted one way ANOVA was conducted. Significant differences were evaluated if ANOVA was significant. The significance of individual differences was evaluated using the Bonferroni *t* method. A *p*-value of less than 0.05 was considered to be statistically significant.

## Results

**Figure 1:**
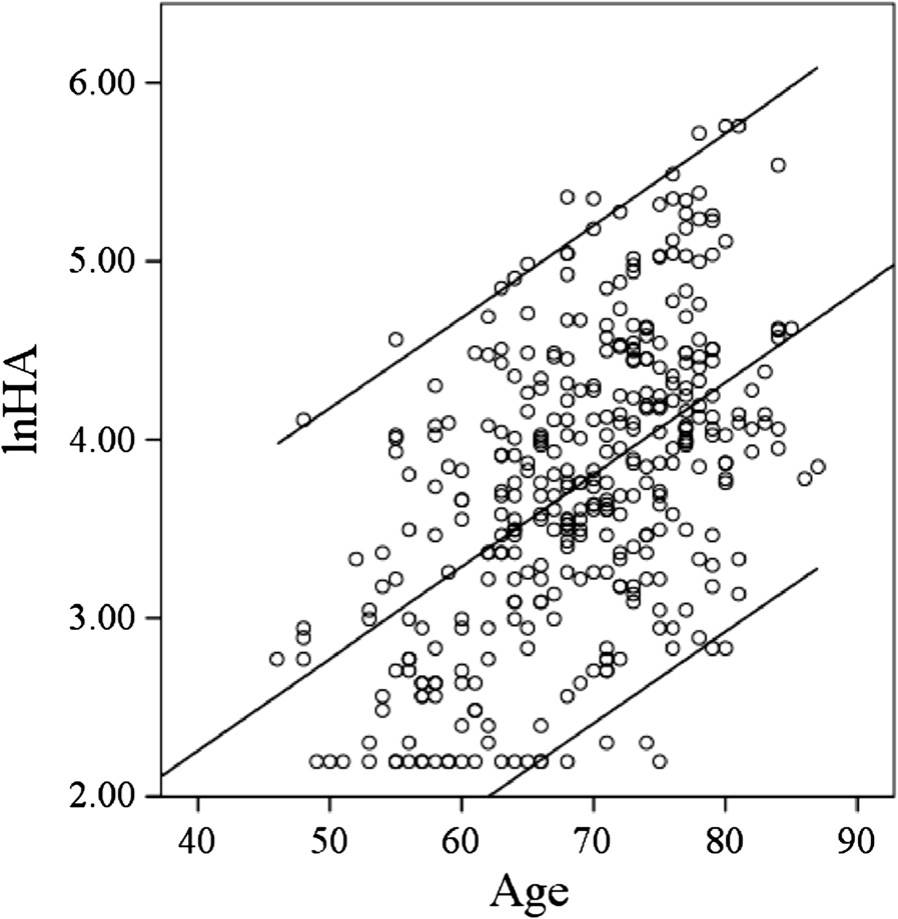
Correlation between the sHA levels and age of the patients. ln-sHA; logarithmically transformed sHA. n=372.

The age of the patients was significantly increased depending upon the progression of the disease (K/L grades**)**(r=0.46, Figure 1), as reported previously [7]. The BMI of the patients with either K/L grade 1 or 2 were significantly different from those with K/L grade 3 and 4, respectively.

The ln-sHA levels of the subjects with K/L grade 4 were significantly increased in comparison to those with K/L grade 1, 2 and 3, respectively (Table 1). On the other hand, no significant difference of the ln-sHA levels were observed between the subjects with K/L grade 3 and those with K/L grade 1 and 2 (Table 1). There were no significant differences of the ln-sHA levels between the subjects with K/L grade 1 and grade 2 (Table 1).

**Table 1:** Logarithmically transformed sHA (lnHA) levels of female patients with knee OA corresponding to the K/L grade

**K/L**	**1**	**2**	**3**	**4**	***p*for trend**
LnHA	3.50 (0.11, 3.28-3.72)	3.55 (0.08, 3.39-3.71)	3.74 (0.08, 3.58-3.89)	4.05 (0.08, 3.90-4.20)	<0.001
		* *p*=1.00 (−0.41 to 0.30)	* *p*=0.47 (−0.60 to 0.12)	* *p*<0.001 (−0.92 to −0.19)	
			*¶p*=0.60 (−0.49 to 0.11)	*¶p*<0.001 (−0.80 to −0.20)	
				† *p*<0.05 (−0.60 to −0.03)	

All analyses were adjusted for age and body mass index (BMI). Covariates appearing in the model are evaluated at the following values; age = 69.1, BMI = 24.5. A *p* value less than 0.05 was considered to be significant. Data indicates mean (SD, 95%CI) and *p* (95%CI for differences). K/L: Kellegren and Laurence grade. *: vs. K/L 1, ¶: vs. K/L 2, †: vs. K/L 3.

The means and 95% confidence interval (CI) of ln-sHA were reversed into constant sHA and the age and BMI adjusted reference intervals for sHA corresponding to the patients with K/L grade 1 to 4 were calculated (Table 2). The reference intervals for sHA corresponding to the patients with K/L grade 1, 2 and 3 overlapped considerably. However, the reference interval of sHA corresponding to the patients with K/L grade 4 did not show any overlap between those with any other grades (Table 2).

**Table 2:** sHA reference intervals of female patients with knee OA corresponding to the K/L grade

	**K/L**	**1**	**2**	**3**	**4**
sHA	Mean	33.0	34.8	42.0	57.4
(ng/ml)	(SD)	(2.1)	(2.1)	(2.0)	(2.1)
	Reference interval	26.5 - 41.1	29.7 - 40.8	36.0 - 48.9	49.6 - 66.5

Data indicates mean (SD). All analyses were adjusted for age and body mass index (BMI). Covariates appearing in the model are evaluated at the following values; age = 69.1, BMI = 24.5. K/L: Kellegren Laurence grade.

## Discussion

No biomarkers have so far been established as accepted tools for characterizing the status of OA [12]. This is particularly due to the fact that there are no biomarkers in OA that can characterize the disease state in an individual patient [3,13]. In addition, the burden of disease markers can be used only for clinical studies because individual values obtained in groups of patients with different degrees of OA burden overlap considerably [14]. A reexamination of the approach for reporting biomarker results was proposed to overcome this problem [12], and this study was conducted to determine whether sHA levels could be employed as a burden of disease marker for individual patients by estimating the reference intervals of sHA levels corresponding to the radiographic severity of knee OA. The results revealed that the reference intervals of sHA in patients with severe knee OA (K/L grade 4) can be estimated without overlap, while those of the patients with primary to moderate OA (K/L grade 1 to 3) overlapped, considerably. These findings indicate that sHA can be available as a burden of disease marker for severe (K/L grade 4) knee OA patients and cannot for primary to moderate (K/L grade 1, 2, and 3) knee OA patients.

A measured or observed laboratory test, such as biomarker, are established when a person (usually a patient) is compared with a reference interval for the purpose of making a medical diagnosis, therapeutic management decision, or other physiological assessment. The interpretation of clinical laboratory data is, therefore, a comparative decision-making process. This decision making process requires reliable reference intervals for the biomarkers [8]. Therefore, the establishment of the reference intervals for sHA may be useful to predict the prognosis of the disease in an individual patient, because sHA has been reported to be a biomarker that can predict the prognosis of the disease [15,16], although further study is necessary.

The burden of disease biomarkers indicates the extent or severity of disease, and therefore such biomarkers can be considered to be useful tools for the staging of the disease [13]. The sHA level has been reported to be a biomarker of radiographic knee OA [7]. In general, the severity of OA increases with age, and the sHA level was increased with age in our study (Figure 1). It is impossible to exclude the possibility that an overlap of the reference intervals for the sHA levels corresponding to the patients with K/L grade 1, 2 and 3 may be due to the influence of the extent of the disease in other joints [2]. However, even though the level in patients with K/L grade 4 must be affected by the extent of the disease in other joints, no overlap was shown for the reference interval of the sHA level corresponding to the patients with K/L grade 4. The sHA level has been reported to be a biomarker not only of the burden of disease, but also as a marker of disease progression [16]. Therefore, the result of this study could be meaningful, as we were able to identify the patients with progressive OA using the sHA level, in addition to examining the radiographic severity of OA because we cannot distinguish the patients with progressive OA from those without using solely radiography [3,5]. For example, the sHA, in addition to the radiography, may be helpful to identify patients with progressive OA for inclusion into future clinical trials of DMORDs.

This current study had some limitations. The study included only Japanese female patients in the analyses. Therefore, the findings cannot be generalized to other ethnic groups and male patients. Although sHA levels were affected by total body burden of disease [17], there was no detailed phenotyping of other joints in this study, as mentioned above. Thus, the contribution of other joints to the systemic levels of biomarkers cannot be addressed.

## Conclusions

In conclusion, this study revealed the potential of sHA as a burden of disease biomarker for the evaluation of individual patient with severe knee OA (K/L grade 4) and, simultaneously, revealed the limitation of sHA as a burden of disease biomarker for the evaluation of individual patient with primary to moderate knee OA (K/L grade 1, 2, and 3).

## Abbreviations

OA: Osteoarthritis; NIH: National Institute of Health; OARSI: Osteoarthritis Research Society International; BIPEDS biomarker classification: Burden of disease, Investigative, Prognostic, Efficacy of intervention, Diagnosis of the Disease and Safety of interventions; sHA: Serum level of hyaluronic acid; IFCC: International Federation of Clinical Chemistry and Laboratory Medicinel; CLSI: Clinical and Laboratory Standards Institute; ACR: American College of Rheumatology; K/L: Kellegren and Laurence; BMI: Body mass index; ANOVA: Adjusted one way analysis of variance; ln-sHA: Logarithmically transformed sHA; CI: Confidence interval.

## Competing interests

The authors declare that they have no competing interests.

## Authors’ contributions

HK and MI have made substantial contributions to conception, design, acquisition of data, analysis and interpretation of data, and drafting the manuscript. TD has made contributions to conception, design, analysis and interpretation of data. IF, SH, TK, MK, YS, YT, and HI have made contributions to acquisition, analysis and interpretation of data. LL, RS and AY have made contributions to analysis, interpretation and statistical analysis of data. HK and KK have made contributions to conception, design of data and been involved in drafting the manuscript. All the authors also have given final approval for submitting the manuscript.

## Pre-publication history

The pre-publication history for this paper can be accessed here:

http://www.biomedcentral.com/1471-2474/14/34/prepub
